# Crystal Protein of a Novel *Bacillus thuringiensis* Strain Inducing Cell Cycle Arrest and Apoptotic Cell Death in Human Leukemic Cells

**DOI:** 10.1038/s41598-019-45928-z

**Published:** 2019-07-04

**Authors:** V. Beena, V. Ramnath, K. P. Sreekumar, K. Karthiayini, P. T. Philomina, D. Girija

**Affiliations:** 1 Department of Veterinary Physiology, College of Veterinary and Animal Sciences, Mannuthy, Kerala Veterinary and Animal Sciences University, Thrissur, Kerala India; 2 Department of Agricultural Microbiology, College of Horticulture, Kerala Agricultural University, Vellanikkara, Thrissur, Kerala India

**Keywords:** Cancer, Drug development

## Abstract

Parasporal inclusions of a native non haemolytic *Bacillus thuringiensis* strain KAU 59 was screened for its cytotoxicity against human lymphocytic leukemic cell line jurkat and normal human lymphocytes. The cytotoxicity of proteinase activated and non activated solubilised parasporal inclusions against both cell lines was assessed by Cell Titer 96 Aqueous Non Radioactive Cell Proliferation Assay Kit using MTS. The 50 per cent effective concentration (EC_50_) values were deduced from log probit analysis at 48 h. Morphological changes associated with cytotoxicity were evaluated and molecular mechanisms of cell death were elucidated by TUNEL assay at 48 h post-inoculation. The fluorescence assisted cell sorting was done in the flow cytometer to assess the stage of cell cycle arrest. Relative quantification of caspase-3 expression in Jurkat cells treated with parasporal inclusion protein of KAU 59 was done by qRTPCR The results indicated that the protein was cytotoxic to jurkat cells at the same time non toxic to normal lymphocytes. Cytotoxicity was evident only after proteolytic activation. Apoptotic cell death was confirmed in the protein treated cells by TUNEL Assay and also up regulated caspase-3 gene expression (P < 0.001). S phase cell cycle arrest was confirmed by and fluorescence associated cell sorting.

## Introduction

*Bacillus thuringiensis* (Bt), a member of the genus Bacillus, is a rod shaped, motile Gram-positive, facultative anaerobic and spore forming soil bacterium. When nutrients and environmental conditions are sufficient for growth, the spore germinates producing a vegetative cell that grows and reproduces by binary fission. When the growth conditions become unfavourable, it produces the dormant endospore which is resistant to organic solvents, inactivation by heat and desiccation. Formation of crystal (Cry) proteins encoded by Cry genes of plasmids adjacent to the endospore is the key function discriminating Bt from related species^1^ and the toxic activity of Bt is attributed to these Cry proteins The remarkable diversity of Bt strains and toxins are due to a high degree of genetic plasticity. The protein accumulation in the mother cell compartment form crystal inclusion that could account for 20 to 30 per cent of the dry weight of the sporulated cell^2,3^. The parasporal inclusions of Bt contained ∂ endotoxins which were reported to be specifically toxic to agriculturally and medicinally important insect pests of several orders^1^. These proteins are produced as crystal inclusions adjacent to the endospore as inactive pro toxins. The protoxins dissolved in the alkaline environment of the midgut of the insect larvae, digested by specific proteases to form active toxins^4^ which form pores in the epithelial membrane.

Researches on the biological activities of Bt strains with non-insecticidal parasporal inclusions, which are abundant in nature had led to the discovery of a unique group of proteins called ‘Parasporins’. They are the crystal proteins of *Bacillus thuringiensis* (Bt) having preferential cytotoxicity against mammalian cancer cells and are non toxic to normal cells^5^. Globally six different PS types had been identified from countries like Japan, Vietnam and Canada. Reports on parasporins with varying cytotoxicity spectra are coming from India and Caribbean Island indicating the global dispersion of Bt strains producing the cancer killing toxins. Like the insecticidal cry proteins these proteins are also produced adjacent to the endospore as inactive pro toxins. After extraction they have to be alkali solubilised and proteolytically activated to become active toxins^6^. Though the solubilisation and proteolytic processing remain more or less the same for all parasporins, their cytotoxicity spectra and the modes of cytotoxicity vary with different toxins. The same toxin showed preferential cytotoxicity when treated with different cell lines^7–10^ and most of them were non toxic to normal cells.

Haematological malignancies are charecterised by the presence of increased number of abnormal progenitor cells with diverse stages of haematopoietic differentiation and defective self renewal process in blood and/or bone marrow^11–13^. Since the failure of apoptosis to protect genome integrity during an exposure to oncogenic stimuli is considered as a major reason of such conditions, current approaches for treatment are based on the administration of agents targeting DNA and at the same time with least chemotherapeutic resistance and serious side effects^14^. In this context the identification of novel parasporins and elucidation of their mechanisms of cytotoxicity would be useful in a great extend for the development of promising therapeutic agents in future.

Among the cytotoxic proteins some were proved to be inducing necrotic cell death of tumour cells and caused leakage of cellular contents^15–21^. A proteolytically processed peptide from Bt strain 89-T-34-22 induced, necrosis like cytotoxicity against MOLT-4 cells characterised by mitochondrial swelling and structural breakdown, disorganisation of Golgi complex, cell ballooning and chromatin condensation^15,16^. These changes started 45 min post-inoculation. They could not detect any apoptotic evidence like formation of apoptotic body and DNA fragmentation in susceptible MOLT-4 cells. The toxins can attach to specific receptors on the plasma membrane of the target cells to form an oligomer which can insert itself to the plasma membrane to cause lethal damage to the cells by altering the membrane permeability and efflux of cellular proteins^17,18^. Caspase activation, chromosomal DNA fragmentation and mitochondrial leakage of cytochrome C were not observed even at higher doses of these proteins suggesting the non-apoptotic cell death of the target cells.

There are reports of Bt crystal proteins inducing apoptotic cell death in cancer cells^8,9,22–27^. Some of the reports are based on the cytopathy caused by the toxin on the treated cells such as cytoplasmic granulation and cell ballooning^8,9,23^. Protein from a Malaysian Bt strain, Bt 18 could cause phosphatidyl serine externalisation, activation of caspase-3 and S-phase cell cycle arrest in leukemic cell lines: CEM-SS, CCKF-SB and CCRF-HSB-2^26,27^. These changes were found to be initiated by binding of the toxin with glyceraldehyde 3 PO4 dehydrogenase (GAPDH) present on the plasma membrane of the target cell^22,28^. Crystal protein from another Bt strain KAU 41^29^ was causing cell death by inducing intrinsic pathway of apoptosis in HeLa cells as evidenced by increased expression of apoptotic genes, caspase 3, 9 and Apoptotic Proteases Activating Factor I (APAF I) with no effect of caspase 8.

Extensive screening of native Bt strains for their anticancer properties are undergoing in various parts of the world. Reports clearly indicate that there is wide variation in the cytotoxicity profile of various Bt strains isolated from different geographical locations. Elucidation of the molecular mechanisms of target cell specificity and cytotoxicity of these parasporins will be of great help in designing target specific drugs for cancer therapy and also for differential diagnosis of cancer. In the present study, a non- haemolytic Bt strain KAU 59 isolated from Western Ghats- a biodiversity hot spot of India- was used and the crystal protein isolated from this strain, was tested for cytotoxicity by cell proliferation assay against cell line – Jurkat and also against normal lymphocytes separated from healthy individual. The evidence of apoptotic progression and the stage of cell cycle arrest were assessed in cytotoxic cells by flowcytometry. The relative expression of caspase-3, the executioner caspase of apoptotic pathway, was estimated by q RT-PCR for further confirmation of the apoptotic progression.

## Results

### One dose cytotoxicity assay

The results of one dose haemolytic assay showed that the protein isolated from the Bt strain KAU 59 was non-haemolytic to human erythrocytes. Screening for cytotoxicity by one dose cytotoxicity assay using MTS after 24 h of incubation revealed that the proteolytically activated protein showed mild cytotoxicity to Jurkat cells (13% when compared to mock inoculated control) and non toxic to normal lymphocytes. The level of cytotoxicity to jurkat cells had increased to about 50 per cent and remained non toxic to normal cells when incubated for 48 h. When the incubation period has extended to 72 h about 92 per cent cell death observed with the jurkat cells.

Proteolytic activation is essential for the cytotoxic effect of parasporin. So to check whether the protein possesses all properties of parasporins, cytotoxicity assay was done with non-haemolytic Bt proteins of KAU 59 in intact state (without adding proteinase K). Observations were taken 24 and 48 h post-inoculation. The non activated protein was not only found to be non toxic to Jurkat cells, but increased the cell viability also. This result also proved the effectiveness of proteolytic activation by proteinase K in bringing about the cytotoxicity.

### Dose-response study

**Figure 1:**
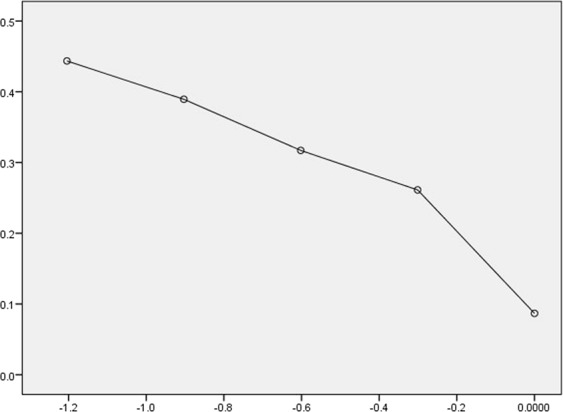
Dose – response curve of cytocidal activity of parasporal inclusion protein of KAU 59 on Jurkat cells 48 h post-inoculation.

The results of the Dose-response study indicated that the EC_50_ value for inclusion proteins of KAU 59, 48 h post-inoculation was 2.913 µg/mL at 95 per cent confidence level (Fig. 1). The value was deduced from the respective dose-response analyses.

### Cytopathy

**Figure 2:**
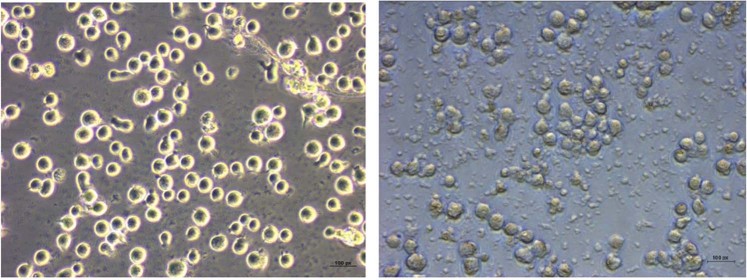
Cytopathic changes of jurkat cells 48 h post-inoculation.

**Figure 3:**
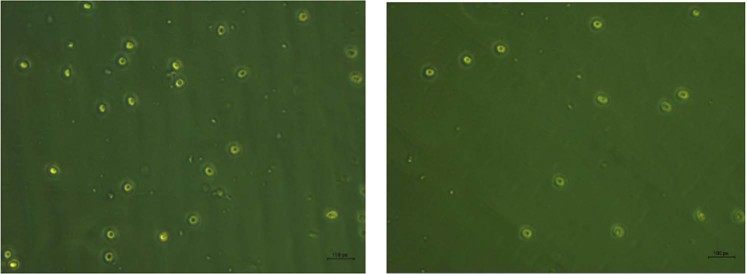
Cytopathic changes of normal lymphocytes 48 h post-inoculation.

The cytopathic changes suggestive of apoptotic progression like shrinkage of cells and membrane blebbing could be detected in jurkat cells after 48 h in the protein inoculated wells (Fig. 2) whereas no changes could be observed in the mock inoculated control cells. At the same time in protein inoculated cells no evidence of cell ballooning was there. Morphological assessment of normal lymphocytes 48 h post-inoculation supported the results of one dose cytotoxicity assay (Fig. 3). No evidence of cell damage could be detected.

### Molecular mechanisms of cell death

**Figure 4:**
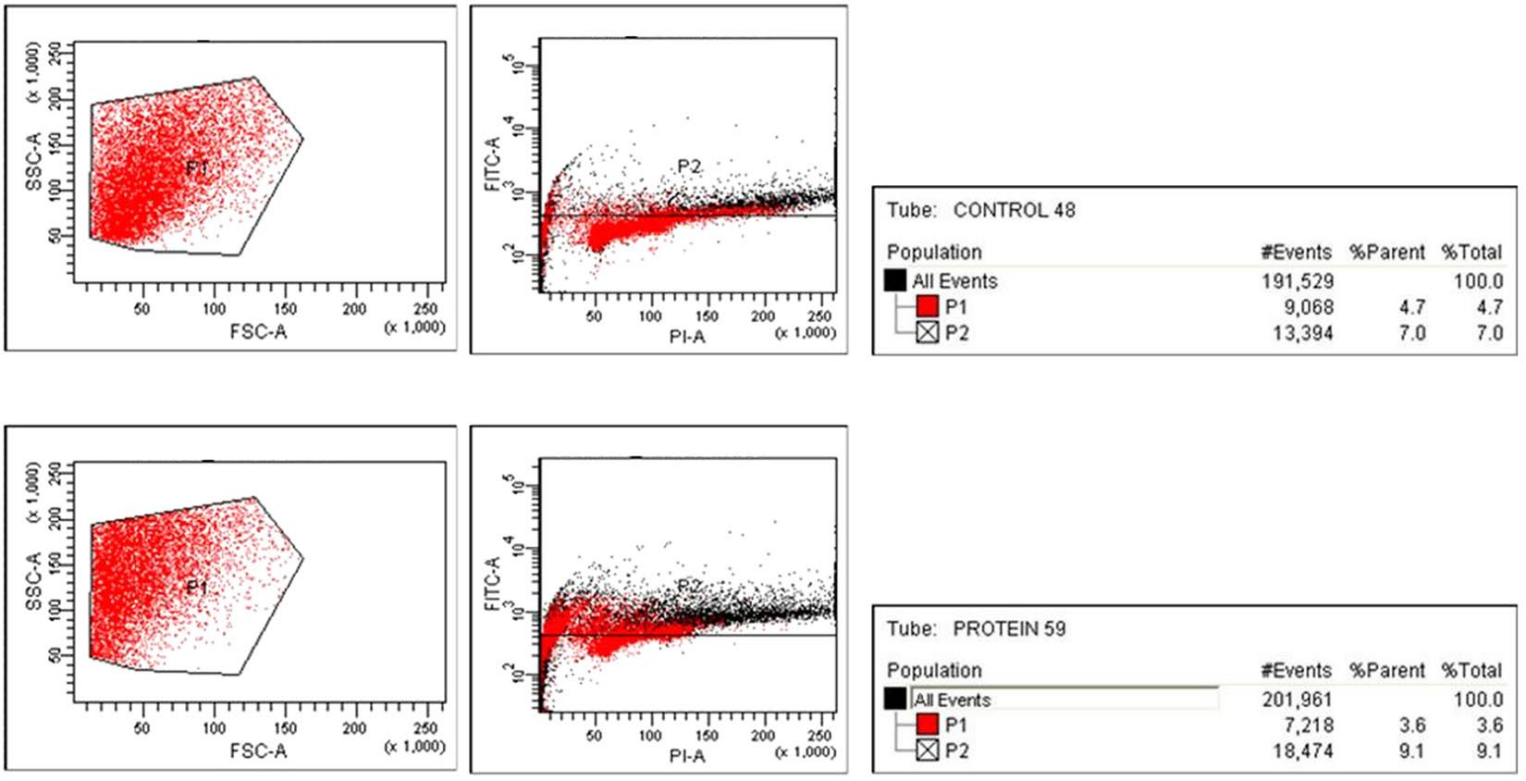
Tunel assay.

**Figure 5:**
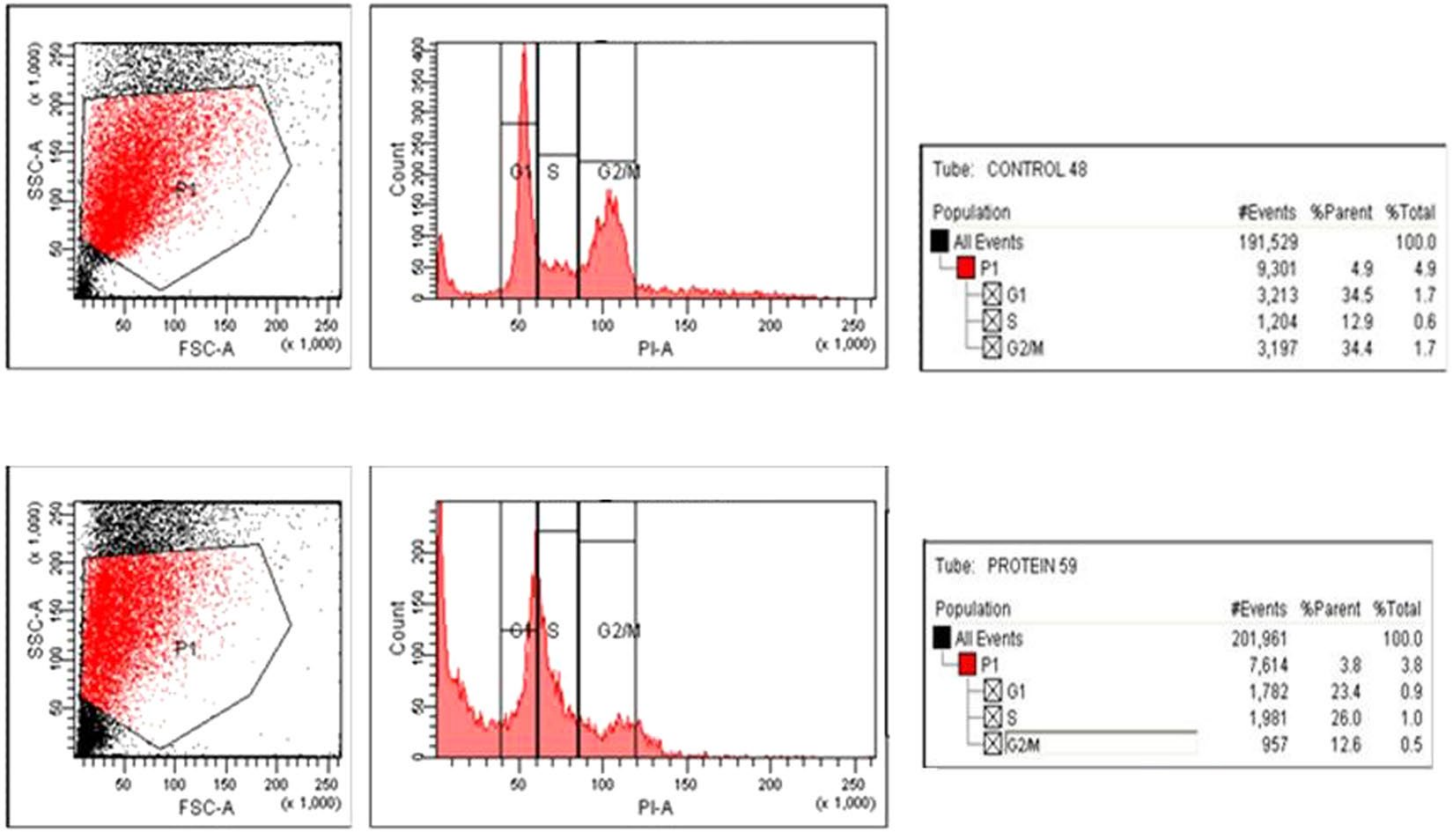
Cell cycle analysis.

Elucidation of molecular mechanisms of cell death caused by the protein on jurkat cells was done by TUNEL assay and real time PCR to quantify caspase-3 expression in Jurkat cells. The result of the TUNEL assay is shown in (Fig. 4). Apoptotic cell death was evident by the increase in FITC tagged cells in the protein treated jurkat cells. The result of the cell cycle arrest analysis is shown in (Fig. 5). The proteins treated Jurkat cells showed cycle arrest at the ‘S’ phase.

**Figure 6:**
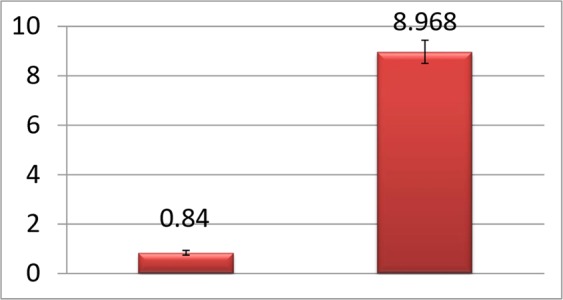
Relative quantification of caspase-3 expression in Jurkat cells.

Relative quantification of caspase-3 expression in Jurkat cells treated with parasporal inclusion protein of KAU 59 revealed 10.7 times (P < 0.001) up regulation of caspase-3 gene expression (Fig. 6).

## Discussion

When one dose cytotoxicity assay was done by adding solubilised and activated inclusion proteins of KAU 59 to 2 × 10^4^ Jurkat cells at 24 h, 48 h and 72 h levels of cytotoxicity were 13 per cent, 50 per cent and 92 per cent respectively. It was also observed that the intact protein (without proteolytic activation) was incapable of inducing cell death in the jurkat cells and normal cells at 24 h and 48 h. At the same time the protein increased the cell viability also indicating the effectiveness of proteolytic activation by proteinase K at the selected dose.

Parasporins are normally non-toxic to normal cells. Whether this property is possessed by the identified cytotoxic proteins of the present study was being assessed by one dose cytotoxicity assay of solubilised and activated inclusion proteins of KAU 59 on normal lymphocytes 48 h post-inoculation. From the results it was clear that the inclusion proteins toxic to leukemic cells were non toxic to normal lymphocytes.

A Bt protein to be included in the group of ‘Parasporins’, should be non-toxic to normal mammalian cells, non-haemolytic to mammalian erythrocytes and becomes toxic to mammalian cancer cells only on proteolytic activation^5,9^. Since the protein of KAU 59 was non haemolytic and becomes cytotoxic only on proteinase K activation, it can be inferred that the anticancer protein of KAU 59 would be a member of parasporin family.

Reports on Bt proteins induced cytotoxicity on Jurkat cells are very less. A cry protein P29 from Bt strain A1519 was reported to possess potent cytotoxicity against Jurkat cells^22,30^ when incubated for 20 h. Although the purified PS1 protein isolated from Bt strain A1190 showed broad spectrum of cytotoxicity, it was non toxic to Jurkat^23^. The proteins PS2Aa isolated from strain A1547 and PS2Ab from Bt strainTK-E6 were reported to be cytotoxic to Jurkat cells^19,31^ within a time span of 2 h. Here the mechanism of cytotoxicity was attributed to the pore forming property of proteins. In the present study, since protein added to medium containing cells could not be remained intact for more than 24 h, it can be inferred that the delayed cell death induced would be the result of a slow death signalling pathway. Cytotoxicity at a later period of 72 h post-inoculation could be due to secondary necrosis. In the *in vivo* system, during apoptosis, early exposure of surface signals will make the scavenger phagocytes to recognize apoptotic cells as unwanted cells. Before the completion of apoptotic programme, the cells are removed and dismantled through heterolysis by phagocytes, thus causing the silent elimination of cells through physiological apoptosis. In the *in vitro* system, in the absence of scavengers, secondary necrosis is activated, at the end of the full apoptotic programme and leads to the autolytic disintegration of the doomed cell. Initiation of secondary necrosis marks the completion of apoptotic pathway.

Like insecticidal cry proteins, conformational change associated with proteolytic activation is essential for the parasporins to cause cytotoxicity of tumour cells. In the present study also the non activated protein was found to be non toxic to the cells. Bt strain 89-7-34-22 was reported to show *in vitro* cytotoxicity to human cancer cells only when activated by proteinase K^15^. Proteolytic activation increased the cytotoxicity of pro-parasporin 1 protein (1 mg/mL) up to a level of 60 µg/mL of proteinase K and above that it sharply decreased^23^ cytotoxicity. Soluble crystal protein preparations of Bt strain MOI9 were found to be non toxic to HeLa unlike their tryptic digests^8^. Mode of cytotoxicity was well explained in some parasporins as the insertion of pore forming domain in to the cell membrane of mammalian cancer cells causing colloid osmotic swelling and cell death^15–17^. Although the molecular reason remain unexplained, proteolytic activation in parasporins causing non necrotic cell death was also found to be essential for inducing tumour cell cytotoxicity^8,10,23^. In the present study, since the primary mode of cytotoxicity observed was not of a necrotic type further studies are needed to identify the receptor mediated molecular events initiating cell death.

On evaluation of cytopathic changes membrane blebbing and cell shrinkage were very much evident at 48 h post inoculation with the protein. These are the characteristic cytopathic changes of apoptotic progression. At the same time cell ballooning was absent indicating the absence of necrosis. Whereas no cytopathic alterations could be observed when normal lymphocytes are incubated with protein.

The cell death of Jurkat cells induced by parasporal inclusion proteins of KAU 59 was found to be apoptosis by TUNEL assay. The per cent of fluorescein isothiocyanate (FITC) tagged cells in control was 7.0 l of parent population, whereas the per cent of the same in KAU 59 treated samples were found to be 9.1 (Fig. 3) indicating a higher level DNA fragmentation in protein treated cells.

The fluorescence assisted cell sorting was done in a flow cytometer to assess the stage of cell cycle arrest caused by the proteins to induce apoptosis. The analysis revealed that the protein caused cell cycle arrest at the ‘S’ phase of the cell cycle.

The cyclical events of cell cycle constituted by cell division, DNA replication and cell growth has mainly 2 major events – an interphase and a mitotic phase. Out of the 3 phases of the interphase, in the first phase ‘G1’, the cell monitor the environment and when the requisite signals are received it synthesise RNA and proteins to induce growth. In the 2^nd^ ‘S’ phase, synthesis and replication of chromosomal DNA takes place. In the 3^rd^ ‘G2’ phase, proteins are synthesised in preparation for mitosis while continuing the growth of cells. After G2 phase, actively dividing cancer cells with diploid number of chromosomes and with double the amount of DNA enter into mitotic cell division, just like a normal somatic cell. If an agent could arrest the sequential event of cell cycle in a cancer cell at any point of cell cycle and lead the cell to undergo physiological death, that agent could become a potential candidate for cancer research. If that cell cycle arrest occurs before DNA replication is completed, the transfer of aberrations on genetic make up to the next progeny cells will be prevented.

In this regard, the Bt protein tested has promising anticancer properties. Cells arrested in the ‘S’ phase of cell cycle was 26 per cent of the parent population for KAU 59 protein inoculated samples of Jurkat cells. In the case of mock-inoculated control the value was 12.9 per cent.

Although apoptotic progression in cancer cell lines, treated with solubilised and activated parasporal inclusion proteins of different Bt strains had been reported, most of them were based on morphological and ultra structural evaluation. The Bt 18 parasporal protein when treated with the leukemic cell lines, the cell cycle was arrested at the ‘S’ phase^27^. In the present study, growth inhibition and induction of apoptosis were observed within 48 h of treatment of cells with proteins.

KAU 59 treated Jurkat cells did not show any evidence of secondary necrosis on 48 h post inoculation. So as a method of confirmation, qRTPCR for caspase-3 expression was also done with KAU 59 parasporal inclusion protein treated Jurkat cells. Caspase-3 is an executioner Caspase of both intrinsic and extrinsic pathways of apoptosis^32^. Nuclear changes associated with apoptosis like chromatin condensation and DNA fragmentation are caused by Caspase-3. Caspase-3 activated DNases are ultimately causing these effects. Caspase-3 expression was found to be 10.7 lines (P < 0.001) up regulated in the protein treated samples compared to mock-inoculated control (Fig. 6). Since DNA fragmentation was evident in TUNEL assay, the significant up regulation of Caspase-3 expression in the samples treated with the protein KAU 59 also conclusively proved apoptosis in Jurkat cells. Up regulation of apoptotic genes, Caspase-3, 9 and Apoptotic Proteases Activating Factor (APAF) I in the parasporal protein of KAU 41 treated HeLa cells were also reported^29^ supporting the evidence of apoptotic cell death in parasporin induced cytotoxicity.

In conclusion this study suggests that the parasporin isolated from the Bt strain KAU 59 causes S phase cell cycle arrest and apoptotic cell death in human lymphocytic leukemic cell line jurkat. Absence of cytotoxicity against normal mammalian cells and the non haemolytic property may make it a potential candidate for cancer therapy in future. Identification of the structure of the protein and its binding properties may help in future for designing anti cancer drugs targeting the specific receptors on cancer cell without eliciting generalised immune responses. Extensive screening of Bt parasporal proteins from different geographical locations may help to identify different novel strains with varying cytotoxicity spectra and these information may throw light on the evolution of cancer and its molecular reasons. This would also reveal the scope of this protein to be used for the differential laboratory diagnosis of cancer. Future researches for elucidation of the molecular reasons of the cytotoxicity spectra, pathways of cytotoxicity and non toxicity towards normal cells will be of great help in designing new similar drugs with wide therapeutic potentials.

## Materials and Methods

### Isolation and activation of crystal protein

*Bacillus thuringiensis* strain KAU 59 was grown in T3 broth^33^ and incubated at 30 °C with continuous shaking at 200 rpm to induce sporulation. Sporulated cultures were harvested by centrifuging the culture medium at 11,000 × *g* at 4 °C for 10 min. The cell debris from the pellet was removed by washing with 5 mL each 2% Triton X-100 in 0.5 *M* NaCl, followed with 0.5 *M* NaCl and finally with deionized water^34^. Solubilisation of inclusions was done in 50 m*M* sodium carbonate (Na_2_CO_3_) (pH 10.0) containing 1 m*M* ethylene diamine tetra acetic acid (EDTA) and 10 m*M* dithio threitol (DTT) for one hour at 37 °C^6^. Insoluble materials were removed by centrifugation at 11,000 × *g* for 10 min at 4 °C. The supernatant was passed through a 0.45 µm PVDF syringe filter (Whatman, UK). Protein concentration of the supernatant fluid was determined by Lowry’s method^35^ using bovine serum albumin as the standard. The pH of the solubilised protein was adjusted to 8.0 and the protein concentration was adjusted to 2 mg/mL. The protein suspension was then treated with proteinase K (120 µg/mL) for 1.5 h at 37 °C. Phenyl methyl sulfonyl fluoride (PMSF) was added to the solutions at a final concentration of 1 m*M* to stop the proteolytic reaction.

### One dose haemolysis assay

Haemolytic activity was tested on human erythrocytes as described by Saitoh *et al*.^36^ with certain modification. Five mL of human blood was collected from a healthy donor. Two per cent (v/v) suspension of erythrocytes was prepared in 20 m*M*/L Tris buffered saline (pH 8.0). The erythrocyte suspension (100 µL) was mixed with an equal volume of inclusion protein from protein solution containing 2 mg/mL protein in 96 well ‘U’ bottom titre plate. After incubation for 18 h at 27 °C, the mixture was centrifuged at 800 × *g* for 10 min and the supernatant was examined for absorbency at 540 nm (Varioskan Flash Thermo Fisher Scientific, Finland). The assay was repeated at least three times. Haemolytic activity was graded as given by Mizuki *et al*.^5^.

### Culture of cell lines

Lymphocytic leukemic cell line- Jurkat, purchased from National Centre for Cell Sciences, Pune, India and normal lymphocytes were used for the study. Normal lymphocytes were isolated from the blood collected from a healthy donor by density gradient centrifugation using HiSep^TM^ LSM (Himedia Laboratories, Mumbai), low viscosity medium containing polysucrose and sodium diatrizoate adjusted to a density of 1.0770 ± 0.0010 g/mL. Blood (2.5 mL) was collected from a healthy donor using heparin coated vacutainers (BD, USA). Isotonic phosphate buffered saline (5 mL) was added to 2.5 mL of blood to get a final dilution of 1:2. Transferred 2.5 mL of Hi Sep^TM^ LSM to a 15 mL clean centrifuge tube and overlaid with 7.5 mL diluted blood without mixing, aseptically. Centrifuged at 700 × *g* in fixed angle rotor at 20 °C with a break off at room temperature for 15 to 30 min. Majority of the plasma and platelet containing supernatant above the interface band was aspirated. Using a clean Pasteur pipette carefully aspirated the lymphocyte layer along with half of the Hi Sep^TM^ LSM layer below it and transferred to a clean centrifuge tube. An equal volume of isotonic PBS was added to the lymphocyte layer in a centrifuge tube and mixed by gentle aspiration. Centrifuged for 10 min at 20 °C at 400 × g and removed the PBS layer. Jurkat cells and normal lymphocytes were cultured in RPMI 1640 (HiMedia Laboratories, Mumbai) containing 10 per cent foetal bovine serum (HiMedia Laboratories, Mumbai) and Kanamycin sulphate (HiMedia Laboratories, Mumbai) at the rate of 30 µg/mL. The cell lines were incubated in humidified CO_2_ incubator (Thermo Fisher Scientific, USA) at 37 °C and 5 per cent CO_2_ tension.

### One dose cytotoxicity assay

One dose cytotoxicity assay was carried out as described by Mizuki *et al*.^6^ with slight modifications. To each well of a 96 well cell culture plate added 90 µL of cell suspension containing 2 × 10^4^ cells estimated by a Neubeur haemocytometer counting chamber. After pre-incubation for 24 h at 37 °C and 5 per cent CO_2_ tension, 10 µL of the proteinase K (120 µg/mL) activated sample solution (2 mg/mL) was added to the well. The solubilising buffer containing proteinase K and PMSF was used as the mock control. The plates were incubated at 37 °C, 5 per cent CO_2_ tension for 24 h for screening the cytotoxicity. The test was also done with non-activated (without proteinase K added) protein The test was repeated for the cytotoxic protein at 48 h and 72 h of incubation also. The level of cytotoxicity was assessed by Cell Titer 96 Aqueous Non Radioactive Cell Proliferation Assay Kit (Promega, USA)^24^ using A tetrazolium compound [3-(4, 5-di Methyl thiazol-2-yl)-5-(3-carboxymethoxy phenyl)-2-(4-sulfophenyl)-2H-tetrazolium, inner salt; MTS] and an electron coupling agent – Phenazine methosulfate; PMS. Combined MTS/PMS solution (20 µL) was pipetted into each well of the 96 well assay plate containing 100 µL of cells in culture medium. The MTS solution and the PMS solution were thawed by keeping at 37 °C for 10 min and 2 mL of MTS solution taken in a test tube, added 100 µL of PMS solution, just before the start of the experiment and mixed thoroughly. 20 µL of the combined MTS/PMS solution was pipetted into each well of the 96 well assay plate containing 100 µL of cells in culture medium. Incubated the plate for 1 to 4 hours at 37 °C in a humidified incubator at five per cent CO_2_ tension and recorded the absorbance at 490 nm using Varioskan Flash plate reader (Thermo Fisher Scientific, Finland). The average of absorbance values in mock inoculated negative control was used as the blank value. The relative value of absorbance to the blank (1.0) and the percentage change of absorbance of protein treated sample from that of the control was recorded. The tests were repeated at least three times.

### Dose-response study

The cytotoxicity assay was repeated, separately in triplicates for eight different two fold serial dilutions prepared in TED buffer (50 m*M* Tris (pH 9.0) – 1 m*M* EDTA – 2 m*M* DTT). The cytotoxicity was measured by MTS assay and the 50 per cent effective concentration (EC_50_) values were deduced from log probit analysis and dose response curves were plotted.

### Cytopathy and morphological changes

Morphological changes associated with cytotoxicity were evaluated using inverted phase contrast microscope at 48 h post-inoculation.

### Apoptotic detection and cell cycle analysis

Molecular mechanisms of cell death was elucidated 48 h post-inoculation by terminal deoxy nucleotidyl transferase dUTP nick end labelling (TUNEL) assay using ApopTag® Fluorescein *In Situ* Apoptosis Detection Kit (Millipore, Canada) by flow cytometry (BD FACS Aria I, USA. The fluorescence assisted cell sorting was done in the flow cytometer to assess the stage of cell cycle arrest caused by the proteins to induce apoptosis. The results were analysed using the software BD FACSDiva.

### Expression analysis of caspase-3

Relative quantification of caspase-3 expression in Jurkat cells treated with parasporal inclusion protein of KAU 59 was done by qRTPCR. After 24 h pre incubation, the cells were treated with the proteinase K activated toxin with the same dose as in one dose cytotoxicity assay and incubated for 48 h. In the control wells, the cells were incubated for 48 h with solubilising buffer containing proteinase K and PMSF. After the incubation RNA was isolated by TRIzol method. RNA quantification and purity checking were done by nanodrop (Thermo Fisher Scientific, USA). Reverse transcriptase PCR was done in a thermal cycler (Applied Biosystems, USA) for cDNA synthesis using High – Capacity cDNA Reverse Transcription Kit (Applied Biosystems, USA).

Real Time PCR was done to quantify the expression of caspase-3 gene in control and protein treated cells in comparison to housekeeping gene β actin. *Power SYBR® Green PCR Master Mix* (Applied Biosystems, USA*)* was used along with the following primers.

For β actin^37^ - F:CCAGCTCACCATGGATGATG

R: ATGCCGGAGCCGTTGTC

ForCaspase-3^38^-F:TTCAGAGGGGATCGTTGTAGAAGTC

R:CAAGCTTGTCGGCATACTGTTTCAG

The primers were custom synthesised from Sigma-Aldrich, Bangalore. They were diluted with sterile, nuclease free milliQ water to obtain a final concentration of 100 n*M*/µL. The reaction mix was prepared and added at the rate of 20 µL per well of the MicroAmp® Optical 96-well Reaction Plate (Applied Biosystems, USA). Separate PCR reactions were set for each caspase-3 and β actin genes. For the protein and control 3 biological replicates were used. Each sample was amplified in 3 technical replicates. In addition, one non-template control (NTC) for each gene and reverse transcription minus (RT minus) control by loading RNA instead of cDNA for each sample and a negative control (with only nuclease free water) were also included in the reaction. The plate was sealed with MicroAmp® Optical Adhesive Film (Applied Biosystems, USA). The qRTPCR was performed in Real Time PCR system ((Applied Biosystems, USA) as per the programme:- Initial denaturation 95 °C for 10 min, 40 cycles of 95 °C for 15 sec and 60 °C for 1 min. Melt curve was generated using Applied Biosystems real-time PCR system software and confirmed the absence of nonspecific amplification. Thermal cycling for melt curve analysis was 95 °C for 15 sec, 60 °C for 1 min and 95 °C for 15 seconds. Data acquisition is performed during the annealing step. Data were analysed for relative quantification by comparative ∆∆Ct method.

### Statistical analysis

Independent T test was done to compare the fold of expression both in control and treated cells. Statistical significance was established at P < 0.001.

It is certified that all experiments were performed in accordance with relevant guidelines and regulations as approved by the research council of the Kerala Veterinary and Animal Science University. It is also certified that the experiments including the human blood sample collection were approved by the institutional ethics committee with the informed consent of the individual.

## Data Availability

I do here by state those materials, data and associated protocols will be promptly available to readers without undue qualifications in material transfer agreements.
